# Studying de-implementation in health: an analysis of funded research grants

**DOI:** 10.1186/s13012-017-0655-z

**Published:** 2017-12-04

**Authors:** Wynne E. Norton, Amy E. Kennedy, David A. Chambers

**Affiliations:** 1 Division of Cancer Control and Population Sciences, National Cancer Institute, National Institutes of Health, 9609 Medical Center Drive, Bethesda, MD 20850 USA; 2 Center for Research Strategy, Office of the Director, National Cancer Institute, National Institutes of Health, Bethesda, MD USA

**Keywords:** De-implementation, Decrease use, De-prescribe, Disincentivize, Disinvestment, Exnovation, Low value, Portfolio analysis, De-adoption, Medical reversal, Implementation science, Implementation research, Overuse, Evidence-based

## Abstract

**Background:**

Studying de-implementation—defined herein as reducing or stopping the use of a health service or practice provided to patients by healthcare practitioners and systems—has gained traction in recent years. De-implementing ineffective, unproven, harmful, overused, inappropriate, and/or low-value health services and practices is important for mitigating patient harm, improving processes of care, and reducing healthcare costs. A better understanding of the state-of-the-science is needed to guide future objectives and funding initiatives. To this end, we characterized de-implementation research grants funded by the United States (US) National Institutes of Health (NIH) and the Agency for Healthcare Research and Quality (AHRQ).

**Methods:**

We used systematic methods to search, identify, and describe de-implementation research grants funded across all 27 NIH Institutes and Centers (ICs) and AHRQ from fiscal year 2000 through 2017. Eleven key terms and three funding opportunity announcements were used to search for research grants in the NIH Query, View and Report (QVR) system. Two coders identified eligible grants based on inclusion/exclusion criteria. A codebook was developed, pilot tested, and revised before coding the full grant applications of the final sample.

**Results:**

A total of 1277 grants were identified through the QVR system; 542 remained after removing duplicates. After the multistep eligibility assessment and review process, 20 grant applications were coded. Many grants were funded by NIH (*n* = 15), with fewer funded by AHRQ, and a majority were funded between fiscal years 2015 and 2016 (*n* = 11). Grant proposals focused on de-implementing a range of health services and practices (e.g., medications, therapies, screening tests) across various health areas (e.g., cancer, cardiovascular disease) and delivery settings (e.g., hospitals, nursing homes, schools). Grants proposed to use a variety of study designs and research methods (e.g., experimental, observational, mixed methods) to accomplish study aims.

**Conclusions:**

Based on the systematic portfolio analysis of NIH- and AHRQ-funded research grants over the past 17 years, relatively few have focused on studying the de-implementation of ineffective, unproven, harmful, overused, inappropriate, and/or low-value health services and practices provided to patients by healthcare practitioners and systems. Strategies for raising the profile and growing the field of research on de-implementation are discussed.

## Background

In recent years, as healthcare costs have continued to rise [1], wasteful spending has been identified [2, 3], and more robust evidence about health practices and programs has become available, issues pertaining broadly to reducing (frequency and/or intensity) or stopping (ceasing) the use of harmful, ineffective, low-value, and/or unproven health services and practices have become more salient [4–6]. Indeed, overuse of health services and practices is quite costly: a report from the Institute of Medicine (IOM) estimated that waste in healthcare accounted for approximately $750 billion in 2009. Further, Berwick and Hackbarth [2] estimated that overtreatment accounted for upwards of $226 billion in wasteful spending in 2011. Rates of overuse vary widely by health area, patient population, and type of health service or practice [5, 7–9]. Among a sample of 2106 physicians in the US, participants considered approximately 20% of overall medical care to be unnecessary, including prescription medications (22%), tests (24.9%), and procedures (11.1%) [10]. Overuse of health services and practices has a deleterious effect on patients too, including cost, emotional distress, anxiety, harm, physical discomfort, adverse events, incidental findings, and quality of life, among others [10–14].

With the increasing recognition of these issues, there now exist specialty conferences and tracks focused on overuse (e.g., Preventing Overdiagnosis Conference (http://www.preventingoverdiagnosis.net)) and professional society campaigns (e.g., American Board of Internal Medicine’s [ABIM] *Choosing Wisely*; [15]). Moreover, the number of commentaries and empirical studies on medical reversals [16–18], overuse (i.e., including overuse of screening, testing, and treatment) [4, 5, 8], inappropriate or misuse [13], and low-value care [19, 20] is increasing. Publications on de-implementation of specific health services and practices are increasing. Niven and colleagues identified 43 unique terms relevant to de-adoption, operationalized as “the discontinuation of a clinical practice after it was previously adopted,” among a sample of 109 articles [21]. Such variability in the use of terms is similar to that in implementation science, as reported by McKibbon and colleagues [22] in 2010.

Considerations for studying de-implementation, and identification of multilevel, contextual factors that may facilitate or impede de-implementation, have been discussed in the literature. For example, Prasad and Ioannidis described de-implementation processes that may vary as a function of the type of evidence for the practice, including (1) medical practices for which existing evidence is contradictory, (2) medical practices that are unproven, and (3) medical practices that are novel despite widespread use [17]. Importantly, Prasad and Ioannidis point to the need for more rigorous and replicable studies as a prerequisite to justify broader adoption, implementation, and routine use of health services and practices. Montini and Graham explored historical, economic, professional, and societal factors associated with de-implementation using radical mastectomy as a case study [23]. Niven and colleagues outlined ethical considerations for the discontinuation of health services and practices, including issues pertaining to beneficence, non-maleficence, justice, and autonomy [24]. Several studies have focused on understanding factors associated with de-implementation and developing strategies to facilitate de-implementation as well (e.g., [19, 25–28].

To complement ongoing efforts to study de-implementation, we used systematic methods to analyze grants funded by the US National Institutes of Health (NIH) and the US Agency for Healthcare Research and Quality (AHRQ) using a search database. Consistent with the general goals of portfolio analyses, our objectives were to identify and describe research studies on de-implementation. Such data are critical for assessing the current state-of-the-science, synthesizing findings across health areas and delivery settings, and informing targeted efforts needed to advance research in this area.

## Methods

Consistent with best practices in portfolio analyses, we used the NIH internal-use-only Query, View and Report (QVR) system to identify funded research grants on de-implementation across all 27 NIH Institutes and Centers (ICs) and AHRQ. The analysis included a selective text query involving key search terms along with specific criteria to find the most relevant grants. We limited our search to research-specific grants (vs. conference grants, for example), including the R-series mechanisms (research proposals; R01, R21, R03, R56 [29]) and the K-series mechanisms (Career Development Awards, which include training objectives and a study proposal; K08, K12, K23, K24) [29] that were funded between fiscal year 2000 and February 2017. In addition, we reviewed all research grants that were funded by targeted funding opportunity announcements (FOAs, including program announcements (PAs); program announcement with special receipt, referral, and/or review considerations (PARs); and request for applications (RFAs) with at least a partial (but not exclusive) focus on areas related to de-implementation (e.g., implementation science, healthcare delivery).

### Search strategy

We relied on the review by Niven and colleagues [21] to inform our selection of search terms for grants on de-implementation. Table 1 displays the 11 terms that were used in the search, including *choosing wisely* [15], *de-adopt%* [21], *decrease use* [21], *de-implement%* [21, 23], *de-prescrib%* [30, 31], *disincent%* [21], *disinvest%* [32, 33], *exnovat*% [34], *low-value* [13, 19, 20], *medical reversal* [14, 18], and *undiffus*% [35], with the “%” notation capturing all tenses and endings for the given base word. The 11 key words were searched in the abstracts, title, and specific aims of grants; grant documents were subsequently extracted from the QVR system for full text coding.

**Table 1:** Search terms (*n* = 11), definition and/or key reference, and targeted funding opportunity announcements (FOA; *n* = 3)

Search term	Definition and/or key reference
Choosing Wisely	• Initiative that aims to promote conversations between clinicians and patients by helping patients choose care that is supported by evidence, not duplicative of other tests or procedures already received, free from harm, and truly necessary [15]
De-adopt%	• Discontinuation of a clinical practice after it was previously adopted [67]
Decrease use	• Reduce intensity and/or frequency of use [21]
De-implement%	• Abandonment [17] • Reduce (frequency and/or intensity) or stop the delivery of ineffective, unproven, harmful, overused, inappropriate, and/or low-value health services and practices provided to patients by healthcare practitioners and systems [68]
De-prescrib%	• Process of tapering, stopping, discontinuing, or withdrawing drugs, with the goal of managing polypharmacy and improving outcomes [31] • Process of withdrawal of an inappropriate medication, supervised by a healthcare professional with the goal of managing polypharmacy and improving outcomes [30] • Planned and supervised process of dose reduction or stopping of medication that may be causing harm or no longer be providing benefit. The goal of de-prescribing is to reduce medication burden and harm while maintaining or improving quality of life
Disincent%	Niven and colleagues [21]
Disinvest%	• Processes of withdrawing (partially or completely) health resources from any existing healthcare practices, procedures, technologies, or pharmaceuticals that are deemed to deliver little or no health gain for their cost and are thus not efficient health resource allocations [32]
Exnovat%	• Exnovation is the process of removal of innovations that do not improve organizational performance, are too disruptive to routine operations, or do not fit well with the existing organizational strategy, incentives, structure, and/or culture [34] • Removal process at the tail end of the innovation cycle [69] • Exnovation is different from “de-implementation,” “de-adoption,” and “rejection” in that these terms emphasize the strategic and deliberate removal of organizational structures and processes, whereas exnovation focuses on the removal of innovations specifically [34]
Low-value	• Services that provide little to no clinical benefit on average [20] • Low-value care can be defined in terms of net benefit, a function of the expected (though uncertain) benefit and cost for an individual or group, and is assessed relative to alternatives, including no treatment [19] • Potential for harm exceeds the possible benefit [13]
Medical reversal	• Medical reversal occurs when an accepted practice—a diagnostic test, medication, or procedure—is overturned. The practice is not replaced by something better, but shown to be inferior to a preexisting, less intensive, or less invasive one [14, 16] • Medical reversal occurs when a currently accepted therapy is overturned—found to be no better than the therapy it replaced [18] • Current practice shown to be ineffective or harmful [21]
Un-diffus%	• Abandoning established practices [35]
FOA	Title and mechanisms
PAR-16-238^a^	• NIH Dissemination and Implementation Research in Health • R01, R21, R03
RFA-CA-15-008^b^	• Research Answers to the National Cancer Institute’s Provocative Questions (Question 12) • R01, R21
RFA-HL-17-016	• National Heart, Lung, and Blood Institute’s Research Career Development Programs in T4 Implementation Research • K12

Terms listed in alphabetical order. % = searching all tenses of the base word. Terms were searched in the grant title, abstract, and specific aims. Full citations can be found in the References section

*FOA* funding opportunity announcement

^a^Most recent R01 FOA listed. Search includes PARs from all years: PAR-06-039, PAR-07-086, PAR-06-520, PAR-06-521, PAR-10-038, PAR-10-039, PAR-10-040, PAR-13-055, PAR-13-056, PAR-13-054, PAR-16-238, PAR-16-236, PAR-16-237

^b^Most recent R01 FOA listed. Search includes RFAs from all years: RFA-CA-13-024 (group E, question 3), RFA-CA-13-025 (group E, question 3), RFA-CA-15-008 (question 12), and RFA-CA-15-009 (question 12)

In addition to the 11 key words, we included funded grants from three specific FOAs related to the study of de-implementation in our search strategy. Funding opportunity announcements are displayed in Table 1 and include (1) NIH Dissemination and Implementation Research in Health (R01, R21, and R03 mechanisms), (2) Research Answers to the National Cancer Institute’s (NCI) Provocative Questions (R01 and R21), and (3) the National Heart, Lung, and Blood Institute’s (NHLBI) Research Career Development Programs in T4 Implementation Research (K12). We searched all R-series and K-series grants funded from these announcements across all years they were accepting applications.

### Eligibility criteria

We used a sequential process to identify eligible grants for full text extraction and coding and to exclude ineligible grants unrelated to de-implementation. Initially, one author (WN) reviewed all titles and excluded irrelevant grants; a second author (AK) reviewed a randomly selected sample of 10% of the excluded grants for quality control. Next, one author (WN) reviewed the abstract and specific aims of the remaining grants and excluded those considered out-of-scope; again, a second author (AK) reviewed a randomly selected sample of 10% of the excluded grants for quality control. Finally, one author (WN) reviewed the entire research plan of each grant for an explicit focus on de-implementation and excluded those deemed irrelevant. The final sample of grants was coded.

### Codebook development and coding process

The codebook was developed through an iterative process that included review of other NIH-specific portfolio analysis codebooks and publications [36–38], NIH’s Office of Portfolio Analysis [39], review of the literature on de-implementation [17, 21, 23, 35], and discussion among the study team. A randomly selected subset of four grants was double-coded by two authors (WN and AK) to pilot the codebook; the codebook was discussed, refined, and finalized. The final version of the portfolio analysis codebook (Appendix 1) included eight domains and 36 codes with a “select all that apply” response option. Final domains (and select examples of codes) include (1) *overall study objective* related to de-implementation (e.g., understand or characterize factors influencing de-implementation; develop strategies to facilitate de-implementation), (2) *health area* (e.g., cancer, cardiovascular disease), (3) *continuum of care* (e.g., prevention, treatment), (4) *practice or program* (e.g., medication, screening test), (5) *target patient population* (e.g., children, adults), (6) *study setting* (e.g., hospitals, schools), (7) *study design and research methods* (e.g., experimental, observational), and (8) *data source* (e.g., primary, secondary).

### Data analysis

Descriptive data for eligible grants were extracted from the QVR system, including administrative details (e.g., funding institute/agency, grant mechanism, year awarded), awardee information (e.g., principal investigator’s (PI) primary affiliation, institution type), funding information (e.g., total amount of funding awarded [USD], FOA, study section review), and publications associated with each grant (e.g., overall, journal) to characterize the portfolio of grants included in analyses.

Full grant application files were downloaded from the NIH platform that provides electronic access to complete grant files. The final set of grants was coded by two authors (WN and AK) using the final version of the codebook; codes were compared and discrepancies were discussed until consensus was reached, as applicable. Each coder read each grant application twice: first to gain familiarity with the content and second to code. Frequency and descriptive statistics were used to characterize the overall sample of grants by each of the eight domains and 36 codes, respectively.

## Results

### Search and selection of de-implementation grants

**Fig. 1:**
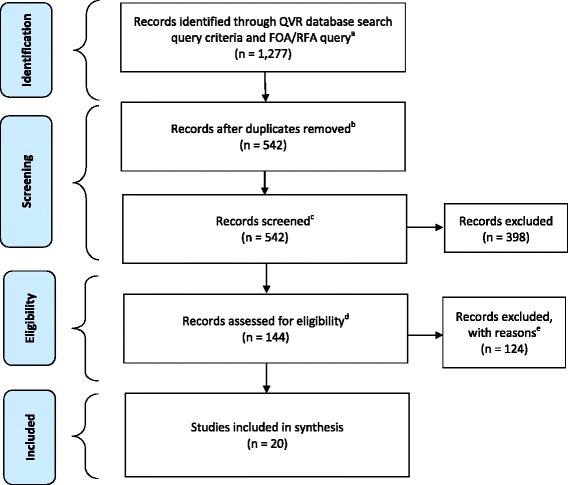
Flow diagram of identification, screening, eligibility, and inclusion of grants for portfolio analysis on de-implementation of health services and practices. Flow diagram adapted from the Preferred Reporting Items for Systematic Reviews and Meta-Analyses (PRISMA) Statement. ^a^Search includes the following: National Institutes of Health and Agency for Healthcare Research and Quality; years 2000–2017; all awarded and funded grants; activity codes for all research grants (R series) and career development awards (K series); free text search in abstract, specific aims, title, and summary statement: disinvest%, medical reversal, de-implement%, de-adopt%, exnovat%, low value, undiffus%, “decrease use,” disincentiv%, “choosing wisely,” and de-prescrib% (combined with “or” and % searching all tenses of the base word). FOA query includes the following: all funded grants from the Dissemination and Implementation Research in Health (DIRH) FOAs, PAR-06-039, PAR-07-086, PAR-06-520, PAR-06-521, PAR-10-038, PAR-10-039, PAR-10-040, PAR-13-055, PAR-13-056, PAR-13-054, PAR-16-238, PAR-16-236, and PAR-16-237. RFA query includes funded grants from the Provocative Questions RFAs, CA-13-024 and CA-13-025 (group E, question 3), CA-15-008, and CA-15-009 (question 12). ^b^Number of unique projects, after removing duplicates (included amended applications, duplicate entries due to multiple principal investigators, etc.). ^c^First found of quality control: examined grant titles and study sections of grants. ^d^Second round of quality control: examined abstract and specific aims of grants. ^e^Exclusion reasons: broad focus on variation in patient outcomes, quality of care, or cost; no specific focus on decreasing or stopping use of health services or practices; and examination of impact of health policy or reimbursement changes on utilization of health services or patient outcomes (e.g., reduction in hospital-associated infections) not specific to de-implementation

Figure 1 displays the Preferred Reporting Items for Systematic Reviews and Meta-Analyses (PRISMA) statement [40] flow diagram for the reporting of systematic reviews and meta-analyses, as adapted to the portfolio analysis. A total of 1277 grants were retrieved using the search terms and targeted FOAs listed in Table 1. After removing duplicates, the titles of the remaining 542 grants were reviewed for general relevance to de-implementation. Of these, 398 grants were deemed unrelated and subsequently removed; example titles of excluded grants include “The Crystal Optimizer: Kinetic Control of Protein Crystallization,” “Role of Mitochondria in HIV Lipoatrophy,” and “Droplet Cell Array Assays.” The abstracts and specific aims of the remaining 144 grants were further reviewed for relevance to de-implementation. A total of 124 grants were excluded, as they did not have an explicit focus on de-implementation. Examples of excluded grants included those proposing to estimate the effect of health policy reform on patients’ utilization of care services, examine factors influencing providers’ use of new effective drugs, and estimate the impact of payment reform on incidence of hospital-associated infections. The remaining grants (*n* = 20) were included in the final sample for the portfolio analysis. A copy of each full grant application was reviewed by two study authors (WN and AK). Titles of the 20 grants are listed in Appendix 2.

### Descriptives of de-implementation grants

Table 2 displays the descriptives of the 20 de-implementation grants included for full coding and analysis. Fifteen grants were funded by NIH and five were funded by AHRQ. Most grants utilized the R-series research mechanism (*n* = 17), including R01 (*n* = 12), R21 (*n* = 3), R03 (*n* = 1), and R56 (*n* = 1), with fewer utilizing the K-series career development award grant mechanism (*n* = 3; K08 = 2; K24 = 1). A little more than half (*n* = 11) were awarded funding between 2015 and 2016, reflecting a marked increase within the past few years. Most were awarded to academic institutions (*n* = 18) compared to a research organization (*n* = 1) or an independent hospital (*n* = 1). Principal Investigators’ (PIs) affiliations were with schools/colleges of medicine (*n* = 18), schools/colleges of public health (*n* = 8), and schools/colleges of pharmacy (*n* = 1). Among the sample of 25 PIs (including co-PIs for five grants), 10 held a medical degree (MD), 12 held a doctoral degree (PhD or ScD), and three held a dual degree (MD/PhD). Per NIH career-phase classification, 10 were new investigators and two were early stage investigators. Table 3 displays the amount of money (USD) awarded for all 20 grants, stratified by R-series or K-series and by specific mechanism. A total of $16.5M in direct costs was awarded for all 20 grants; R-series grants totaled $14.9M and K-series grants totaled $1.5M, respectively.

**Table 2:** Descriptives of de-implementation grants (*N* = 20)

Variable	Total	
	*n*	%
Primary funding agency/institute		
AHRQ	5	25
NCI	7	35
NHLBI	1	5
NIA	3	15
NIAID	1	5
NIDDK^a^	1	5
NIMH	2	10
Grant mechanism^b^		
R-series	17	85
R01: Research Project	12	60
R21: Exploratory/Developmental	3	15
R03: Small Grant	1	5
R56: High-Priority, Short-Term	1	5
K-series	3	15
K08: Mentored Clinical Scientist	2	10
K24: Midcareer Investigator	1	5
Year Awarded^c^		
2000–2004	3	15
2005–2009	0	0
2010–2014	6	30
2015–2016	11	55
Organization type		
Institution of higher education	18	90
Research organization	1	5
Independent hospital	1	5
PI school/college affiliation^d^		
School/college of medicine	18	72
School/college of public health	8	32
College of pharmacy	1	4
N/A	2	8

*AHRQ* Agency for Healthcare Quality and Research, *NCI* National Cancer Institute, *NHLBI* National Heart, Lung, and Blood Institute, *NIA* National Institute on Aging, *NIAID* National Institute of Allergy and Infectious Diseases, *NIDDK* National Institute of Diabetes and Digestive and Kidney Diseases, *NIMH* National Institute of Mental Health, *PI* Principal Investigator

^a^One grant was co-funded with the NIH Roadmap Initiative

^b^Full titles of grant mechanisms: Research Project Grant Program (R01), Exploratory/Developmental Research Grant Award (R21), Small Grant Program (R03), High-Priority, Short-Term Project Award (R56), Mentored Clinical Scientist Research Career Development Award (K08), and Midcareer Investigator Award in Patient-Oriented Research (K24). Details on R-mechanisms: https://grants.nih.gov/grants/funding/funding_program.htm. Details on K-mechanisms: https://researchtraining.nih.gov/programs/career-development

^c^Indicates the first year in which the grant was awarded

^d^Five grants had multiple PIs and four PIs had multiple affiliations. The total number of PI affiliations listed is 29

**Table 3:** Amount of direct costs (USD) awarded for de-implementation grants (*N* = 20)

Grant mechanism	Number of awards	Average cost per grant per year	Total funds
R-series^a^	17	–^b^	$14,985,940
R01: Research Project	12	$398,158	$13,537,372
R21: Exploratory/Developmental	3	$149,492	$896,949
R03: Small Grant	1	$48,500	$97,000
R56: High-Priority, Short-Term	1	$454,619	$454,619
K-series^c^	3	–^b^	$1,565,648
K08: Mentored Clinical Scientist	2	$131,479	$920,350
K24: Midcareer Investigator	1	$161,325	$645,298
Total	20	–^b^	$16,551,588

Reported in USD at time of award. Direct costs only

^a^Full titles of R-series research grants: Research Project Grant Program (R01), Exploratory/Developmental Research Grant Award (R21), Small Grant Program (R03), and High-Priority, Short-Term Project Award (R56). Details on R-mechanisms: https://grants.nih.gov/grants/funding/funding_program.htm

^b^Average cost per grant per year not included for total number of R-series grants, K-series grants, or overall total due to variability in number of years funded for each grant

^c^Full titles of K-series Research Career Development Awards: Mentored Clinical Scientist Research Career Development Award (K08), Midcareer Investigator Award in Patient-Oriented Research (K24). Details on K-mechanisms: https://researchtraining.nih.gov/programs/career-development

Grants were funded under a range of FOAs (Appendix 2). Examples include generally broad FOAs, such as the *AHRQ Health Services Research Projects* (R01 mechanism; PA-13-045; PA-14-291), *AHRQ Mentored Clinical Scientist Research Career Development Award* (K08; PA-13-039), and *Research Project Grant* (R01; PA-13-302), as well as more narrowly focused FOAs, such as *Pilot Clinical Trials for the Spectrum of Alzheimer’s Disease and Age-related Cognitive Decline* (R01; PAR-16-365), *Dissemination and Implementation Research in Health* (DIRH; R21; PA-13-054), and *Research Answers to NCI’s Provocative Questions* (R01; RFA-CA-15-008). Four de-implementation grants were funded through the DIRH FOA, representing 2% of the 201 grants funded through this FOA across the R01, R21, and R03 mechanisms and all years the FOA was available. Interestingly, this was the first time the PIs of these four grants had been funded through the DIRH PAR. Given the range of FOAs, variability was observed in terms of the study sections through which grants were reviewed. For example, three grants were reviewed by the *Health Services Organization and Delivery Study Section* (HSOD) and two each by the *Health Care Research Training Study Section* (HCRT) and the *Healthcare Systems and Value Research* (HSVR), respectively.

Using the bibliometric function in the QVR database, we identified 64 articles that acknowledged at least one of the 20 de-implementation grants as a source of funding (data not shown). Nine grants were associated with at least one publication (mean 7 per grant; range 1–26; median 3). Collectively, the 64 articles were published across 37 journals (mode = 1). Five of the 37 journals published three or more articles, including *Journal of Oncology Practice* (*n* = 7), *Statistics in Medicine* (*n* = 6), *Health Affairs* (*n* = 5), *Health Services Research* (*n* = 3), and *Medical Care* (*n* = 3). When compared to the list of top 20 dissemination and implementation (D&I) journals [41], only seven of those journals overlapped with the 37 journals identified herein. This discrepancy may be due in part to the specialty clinical areas in which the de-implementation grants were funded (e.g., cancer, cardiovascular) compared to the more general areas encompassed in many of the top 20 D&I journals. Of note, however, three of the seven overlapping journals (*Health Affairs*, *Medical Care*, and *Health Services Research*) published three or more articles from at least one of the de-implementation grants, perhaps reflecting a growing recognition that these issues cut across health domains and delivery settings.

Table 4 displays the results of the 20 applications that were reviewed and coded. The overall study objective domain included two codes: (1) understanding, describing, and/or characterizing factors influencing de-implementation and (2) developing, evaluating, and/or testing strategies to facilitate de-implementation, with grants double-coded, where applicable. Among the sample of 20 grants, 14 focused on understanding, describing, and/or characterizing factors influencing de-implementation, and 15 focused on developing, evaluating, and/or testing strategies to facilitate de-implementation.

**Table 4:** Results of portfolio analysis of de-implementation grants (*N* = 20)

Domain	Code	Total	
		*n*	%
Study objectives	Understand or characterize factors influencing de-implementation	14	70
	Develop strategies to facilitate de-implementation	15	75
Health area	Cancer	8	40
	Cardiovascular disease	1	5
	Geriatric syndromes	1	5
	Hormone imbalance	1	5
	Infectious diseases	3	15
	Kidney disease	1	5
	Mental health	2	10
	Neurological	1	5
	Multiple^a^	1	5
	Not specified	1	5
Continuum of care	Prevention	2	10
	Screening and/or detection	5	25
	Diagnosis	3	15
	Treatment	14	70
	Surveillance	2	10
	Not specified	1	5
Health service or practice	Drugs, medications, or therapies	15	75
	Preventive or screening tests	8	40
Target patient population	Children (< 18 years old)	2	10
	Adults (18–64 years old)	12	60
	Older adults (65+ years old)	11	55
Study setting	Clinical care	16	80
	Hospital	4	20
	Nursing homes/assisted living facilities	2	10
	Schools	1	5
Study design and methods	Experimental	7	35
	Measurement/algorithm development	1	5
	Mixed methods	4	20
	Observational	7	35
	Qualitative	3	15
	Quasi-experimental	5	25
	Systems science	4	20
Proposed data source	Primary (e.g., original data collection)	13	65
	Secondary (e.g., claims data)	13	65

Codes were not mutually exclusive. More than one code could be applied to a grant. Numbers may add up to more than 20 (100%) in some cases. Codes were extracted from the text of the full grant application, including abstract, specific aims, and research plan

^a^Multiple: multiple preventive services in primary care settings but health domain not specified

Five grants had an exclusive focus on understanding, describing, and/or characterizing factors influencing de-implementation. For example, one of these five grants proposed to develop and validate a predictive model to identify older, frail, elderly adults for whom colonoscopy would not be recommended, as a first step toward developing a clinical decision support tool for providers to reduce unnecessary colonoscopy procedures. Another grant proposed to use social network analyses to examine multi-level factors associated with the abandonment of inappropriate radiation therapy for cancer patients.

Six grants had an exclusive focus on developing, evaluating, and/or testing strategies to facilitate de-implementation. For example, one grant proposed to conduct a group-randomized controlled trial to assess the impact of an electronic clinical quality measure on reducing overuse of preventive services in primary care settings. Another study proposed to examine the impact of FDA-issued “black box” warning labels and restricted reimbursement on decreasing providers’ use of Epogen to treat anemia in patients with end-stage renal disease. Nine of the 20 grants were coded as having a dual focus on understanding, describing, and/or characterizing factors influencing de-implementation and developing, evaluating, and/or testing strategies to facilitate de-implementation. For example, one grant proposed to study how social networks relate to providers’ recommendation for routine breast cancer screening (including those for whom routine screening is not recommended), and subsequently use agent-based modeling to simulate interventions for changing providers’ screening behavior. Another grant proposed to identify patient- and provider-level factors influencing hormone replacement therapy (HRT) continuation or discontinuation, and to examine temporal trends in HRT prescribing behavior post-Women’s Health Initiative study. As a final example, one grant proposed to understand factors influencing providers’ use of antibiotics for skin and soft tissue infections in the emergency department, and to test the impact of a multi-component antibiotic stewardship intervention on decreasing inappropriate antibiotic prescribing behavior.

The de-implementation grants included a range of both acute and chronic conditions. Although the mode per health area was one, several grants focused on cancer (*n* = 8), mental health (*n* = 2), and infectious diseases (*n* = 3), with most focused on the treatment phase (*n* = 14) in the care continuum. Many grants focused on reducing or stopping the use of drugs, medications, or therapies (*n* = 15; e.g., potentially inappropriate medications (PIMs), including the American Geriatric Society’s Beers Criteria and the Screening Tool of Older People’s Prescriptions (STOPP) criteria [42–44]; non-curative chemotherapy; antibiotics) with comparatively fewer focused on reducing or stopping the use of preventive or screening tests (*n* = 8; e.g., colorectal cancer screening, breast cancer screening, use of imaging and biomarkers for post-treatment surveillance). Grants proposed to study the de-implementation of a health service or practice provided to adults (i.e., 18–64 years old; *n* = 12), older adults (i.e., 65+ years old; *n* = 11), and children (i.e., < 18 years old; *n* = 2). Most proposals focused on clinical settings (i.e., clinical care = 16, hospital = 4, nursing home/assisted living facility = 2), with only one in a non-clinical setting (i.e., school = 1).

A variety of study designs and research methods were proposed across the 20 grants. Somewhat surprisingly, however, given the relatively recent emergence of this field of inquiry, twelve grants (60%) proposed to use an experimental (i.e., randomized controlled trial (RCT), cluster RCT (cRCT), pragmatic RCT (pRCT)) or quasi-experimental (i.e., regression discontinuity, natural experiment, interrupted time series) design in their study. Other grants proposed observational (*n* = 7; prospective or retrospective) or mixed methods designs (*n* = 4).

## Discussion

Relatively few research grants funded by NIH and AHRQ have focused explicitly and/or exclusively on de-implementation, defined as reducing (frequency and/or intensity) or stopping the use or delivery of health services or practices that are ineffective, unproven, harmful, overused, inappropriate, and/or low-value by practitioners and delivery systems to patients. Among the sample of 542 non-duplicative grants, only 20 (3.6%) focused on understanding factors associated with de-implementation and/or testing strategies to facilitate de-implementation. It is encouraging, however, and important to note, that most of these de-implementation grants were funded relatively recently (*n* = 11 in fiscal years 2015–2016), perhaps reflecting the beginning of an upward trend.

The relatively few de-implementation grants identified herein is rather surprising considering the large population of grants from which they were sampled and the fact that we used 11 key search terms and three specific FOAs across all 27 NIH ICs and AHRQ over a 17-year timeframe. In comparison, a recent portfolio analysis on D&I research grants limited to one NIH IC (i.e., NCI) identified 67 funded grants over a 10-year time frame [38]. A separate analysis on D&I research grants funded across nine NIH ICs identified 76 funded grants over a 7-year timeframe [45]. Although uncommon, NIH-wide portfolio analyses have been conducted on single health areas or diseases (e.g., sickle cell disease [46]; cutaneous wounds [47]); such analyses still identified more grants (*n* = 247; *n* = 91) in a shorter time frame (6 years; 1 year) than the 20 grants and 17-year time frame reported herein.

Study findings reflect trends reported in the limited yet growing literature on de-implementation. For example, among the current sample of de-implementation grants, most focused on drugs, medications, or therapies (*n* = 15) and, to a lesser extent, on preventive or screening tests (*n* = 8) in healthcare delivery settings. Consistent with the scoping review by Niven and colleagues [21], as well as literature on prevalence of overuse of health services and practices [8, 48], drugs, medications, and therapies tend to be examined most frequently compared to other services or practices (e.g., behavioral interventions). Future research is needed to understand how strategies for de-implementation may vary as a function of the type of health service or practice (e.g., medical intervention, public health intervention, psychological intervention).

Given the relatively nascent state of the field, a surprising number of grants included experimental or quasi-experimental designs. However, these findings are aligned with a review of published de-implementation studies [21]. Moreover, a systematic review by Tabak and colleagues found that 95 (83%) of studies testing implementation strategies proposed to use an experimental design and 13 (11%) proposed to use a quasi-experimental design [49]. Experimental and quasi-experimental designs are the most appropriate design for testing strategies to facilitate de-implementation, as was the overall objective of many of the de-implementation grants. The range of study designs and research methods proposed in the sample of 20 de-implementation grants is encouraging, to the extent that they reflect the best type of design needed to answer the diverse types of questions in de-implementation. Strategies proposed in the grants overlapped with multilevel classifications identified by Colla [19] and colleagues for reducing the use of low-value services (e.g., patient-, provider-, system-, and policy-level strategies).

Overall, results indicate there is a need for increasing the submission and receipt of research grants on de-implementation within the context of two major biomedical research funding agencies in the US. To increase the number and scope of studies in de-implementation, several targeted efforts on behalf of the research, practice, policy, and funding communities may be warranted. Table 5 summarizes these recommendations and provides examples from other initiatives of how each may be pursued.

**Table 5:** Recommendations for raising the profile of research on de-implementation in health

Recommendation	Examples
1. Raise awareness and interest in studying de-implementation among the research community	• Conferences (e.g., Preventing Overdiagnosis), conference sessions • Webinars, commentaries
2. Develop specific funding opportunities on de-implementation research	• Reducing Overscreening in Breast, Cervical and Colorectal Cancers among Older Adults (PA-17-110)
3. Synthesize and operationalize de-implementation terms, concepts, measures, and outcomes	• State-of-the-art conference • Consensus meetings
4. Collaborate with stakeholders involved in ongoing efforts (e.g., initiatives, campaigns, tools, resources) to study de-implementation in health	• Choosing Wisely® • Canadian Deprescribing Network (CaDeN)
5. Leverage forthcoming policy and practice changes as an opportunity to conduct embedded research on de-implementation	• Oregon Health Insurance Experiment • Opioid crisis in the United Kingdom, Canada, and the United States • Antibiotic resistance crisis

First, as with any emerging area of inquiry, multifaceted approaches are needed to raise awareness and increase interest among the research community, most likely through the traditional forums of conference presentations, publications, meetings, and working groups, some of which are already underway (e.g., [50, 51]). Second, although several funding announcements are available with an explicit focus on de-implementation (e.g., *Reducing Overscreening in Breast, Cervical, and Colorectal Cancers among Older Adults*; R01, PA-17-110; *Dissemination and Implementation Research in Health*; R01, PAR-16-238), additional funding opportunities within and across NIH ICs and AHRQ, as well as other government funding agencies and private foundations, may be needed.

Third, consensus meetings on terminology, definitions, measurement, processes, and outcomes are needed to establish a solid foundation for how best to study de-implementation to move this area of inquiry forward. A recognition of the historical roots of studying overuse, underuse, and misuse, including landmark studies and reports (e.g., [13, 52–55]), as well as the contribution of other disciplines (e.g., clinical psychology, social psychology, public policy, health economics) in understanding and facilitating de-implementation, will serve efforts to advance research in this area well. Consistent with the overall tenets of implementation research, which emphasizes the use of diverse study designs (e.g., experimental, quasi-experimental, observational, modeling), research methods (e.g., qualitative, quantitative, mixed methods), partnerships, context, and generalizability, research on de-implementation may similarly seek to incorporate such perspectives.

Fourth, better coordination with ongoing de-implementation initiatives and key stakeholders is essential for advancing research on de-implementation. Natural partnerships may include those with the *Choosing Wisely* campaign [16] and the Canadian Deprescribing Network [56], among others. Fifth, researchers and funders should leverage forthcoming policy and practice changes as an opportunity to conduct “embedded research” on de-implementation. Embedded research studies are those nested prospectively within ongoing or forthcoming policy and practice changes as such efforts unfold (e.g., Oregon Health Insurance Experiment; [57–59]). Changes in policy—whether it be “small p” policy changes at an organization or “big p” policy changes at local, state, or federal government [60, 61]—may be a particularly opportune time to study how policy termination has an impact on the de-implementation of health services and practices. Across all recommendations, carefully-crafted messages will need to convey the importance and urgency of additional research in this area without inadvertently promulgating misconceptions of withholding appropriate health services and practices.

Several limitations of this study should be noted. Although we used 11 different terms identified from a recent systematic review [21], relevant published literature [14, 17, 35, 62], and targeted FOAs, some grants may not have been captured in our search. In the absence of clear, consensus-based conceptualizations, we relied on published examples of de-implementation protocols and studies (e.g., [25, 27, 63–66]) as well as recent reviews, thought pieces, and conceptualizations of de-implementation [14, 17, 21] to guide our selection of funded grants and subsequent coding process. It is possible that the sample of 20 grants is an underestimate of the total number of grants on de-implementation, to the extent that some grants may include several items in a survey or questions in a semi-structured interview about de-implementation, but do not have a predominant or exclusive focus on de-implementation. Future research is needed to identify similarities and differences between the study of de-implementation of health practices and programs and related areas of inquiry, such as healthcare delivery, implementation science, improvement science, and others. Although related, the predictors, processes, strategies, constructs, and outcomes involved in studying the reduction or cessation of an established health practice or program may be different than those involved in studying the increase or initiation of a new health practice or program, respectively.

Finally, given that our access to full copies of grant applications is limited to two US federally funded research entities (i.e., NIH and AHRQ), these findings may not generalize to other US-funded entities (e.g., Patient-Centered Outcomes Research Institute (PCORI), Centers for Disease Control and Prevention (CDC)) or non-US research-funding organizations.

## Conclusions

Over the past 17 years, relatively few research grants on de-implementation of health services and practices have been funded across NIH and AHRQ. Collaboration is needed among researchers, practitioners, policymakers, patients, and funding agencies to increase the importance of research on de-implementation across health areas, services, practices, and settings. Moving forward, the 20 grants reported herein provide a snapshot of the status of US-funded research on de-implementation and highlight an opportunity for more activity in this area of inquiry.

## Appendix 1

### Portfolio Analysis Codebook

1. What are the overall study objectives? Select all that apply. (Variable: Objectives)
  - Understand, describe, and/or characterize factors influencing de-implementation
  - Develop, evaluate and/or test strategies or approaches to facilitate de-implementation
2. What health domain, condition, disease, or area does the grant focus on? Select all that apply. (Variable: Domain)

- Cancer
- Cardiovascular disease
- Geriatric syndromes (e.g., delirium, falls, functional decline, etc.)
- Hormone imbalance (e.g., hormone replacement therapy)
- Infectious diseases (e.g., urinary tract infections)
- Kidney disease
- Mental health
- Neurological (e.g., Alzheimer’s disease)
- Multiple (e.g., multiple preventive services not specified)
- Not specified

3. What part of the continuum of care does the study address? Select all that apply. (Variable: Continuum)

- Prevention
- Screening and/or detection
- Diagnosis
- Treatment
- Surveillance

4. What is the type of practice, program, intervention, or innovation that is the focus of de-implementation? Select all that apply. (Variable: Practice)

- Drugs, medications, or therapies (e.g., opioids, antibiotics, chemotherapy, dialysis)
- Preventive or screening tests (e.g., mammograms, prostate specific antigen [PSA])
- Other: _____________________________________

5. What is the target patient population (i.e., for whom is the practice, program, intervention, or innovation being de-implemented)? Select all that apply. (Variable: PtPop)

- Children (<18 years old)
- Adults (18–64 years old or non-Medicaid population)
- Older adults (≥65 years old or Medicaid population)

6. In what type of organization, delivery system, or setting does the study take place? Select all that apply. (Variable: Setting)

- Clinical care (e.g., primary care, specialty care)
- Hospital
- Nursing home or assisted living facility
- School (e.g., elementary school)

7. What type of study design and research methods are used? Select all that apply. (Variable: Study)

- Experimental (e.g., randomized controlled trial [RCT], pragmatic RCT [pRCT], cluster/group RCT [cRCT])
- Measurement or algorithm development or validation
- Mixed methods (i.e., collection and integration of qualitative and quantitative data; e.g., sequential exploratory design)
- Observational (e.g., prospective, retrospective)
- Qualitative (e.g., focus groups, semi-structured interviews)
- Quasi-experimental (e.g., regression discontinuity, interrupted time series)
- Systems science (e.g., simulation modeling, social network analysis)

8. What is the proposed source of data collection and analysis? Select all that apply. (Variable: Data)

- Primary data collection and analysis (i.e., research team collecting data for purpose of study; e.g., surveys, focus groups)
- Secondary data collection and analysis (i.e., research team leveraging existing or routinely-collected data for purpose of study; e.g., claims data, chart review, pharmacy refill)

## Appendix 2

**Table 6:** Funding agency, de-implementation grant titles, and funding opportunity announcement (FOA; *N* = 20)

Funding agency	Title	Funding opportunity announcement (FOA)^a^
NIH	1. Behavioral Economics and Improving Chemotherapy Decisions for Advanced Cancer	PA-11-195
	2. Impact of Social Contagion on Physician Use of Unproven Cancer Interventions	RFA-CA-13-024
	3. Targeted Payment Cuts to Reduce Unproven Care	RFA-CA-15-008
	4. A Randomized Controlled Trial to Deprescribe for Older Patients with Polypharmacy Transferred from the Hospital to Skilled Nursing Facilities	PA-14-114
	5. A Statewide RCT [randomized controlled trial] to Reduce Use of Ineffective or Unproven Breast Cancer Care	RFA-CA-13-024
	6. Improving Targeted Colorectal Cancer Screening in the Elderly	PAR-13-146
	7. Measurement of Inappropriate Screening Tests (MIST)	PAR-10-039
	8. Integrated Clinical Prediction Rules: Bringing Evidence to Diverse Primary Care Settings	PAR-13-055
	9. The Impact of Provider Social Networks on Breast Cancer Screening	PAR-13-054
	10. Diffusion of Clinical Evidence into Practice: Physician Networks, Delivery Organizations, and Markets	PA-13-302
	11. HRT [hormone replacement therapy] Decision Making in the Post-WHI [Women’s Health Initiative] Era	Not listed^b^
	12. INtervention for Cognitive Reserve Enhancement in Delaying the Onset of Alzheimer’s Symptomatic Expression: The INCREASE Study	PAR-16-365
	13. Treatment of Anemia in End Stage Renal Disease: Effect of Warnings and Incentives	RFA-RM-11-001
	14. Modeling Treatment Use and Effectiveness in Mental Illness	Not listed^b^
	15. RCT [randomized controlled trial] of TeachTown in Autism Support Classrooms: Innovation and Exnovation	PA-13-216
AHRQ	1. Identifying Cascades of Low-Value Care and the Organizational Practices that Prevent Them	PA-14-291
	2. Improving Antibiotic Stewardship During the Treatment of Skin and Soft Tissue Infections in the Emergency Department: A Human Factors and Systems Engineering Approach	PA-13-039
	3. Reducing Overuse in Primary Care through Safe and Effective Health Information Technology	HS-15-002
	4. Antibiotic Use and Bacteriuria in the Rural Nursing Home	PA-00-010
	5. Variation in Provider Breast Cancer Surveillance Strategies Following Initial Treatment: Contribution of Patient and Provider Factors, Association with Outcomes, and Stakeholder Insights	PA-14-291

The information above is available in the public domain at NIH Research Portfolio Online Reporting Tools (RePORT): https://projectreporter.nih.gov/

*NIH* National Institutes of Health, *AHRQ* Agency for Healthcare Research and Quality

^a^Funding opportunity announcements (number, title): CA-13-024: Research Answers to NCIs Provocative Questions - Group E (R01); CA-15-008: Research Answers to NCIs Provocative Questions (R01); HS-15-002: AHRQ Health Services Research Projects: Making Health Care Safer In Ambulatory Care Settings And Long Term Care Facilities (R01); PA-00-010: Mentored Clinical Scientist Development Award; PA-11-195: Midcareer Investigator Award In Patient-Oriented Research (Parent K24); PA-13-039: AHRQ Mentored Clinical Scientist Research Career Development Award (K08); PA-13-045: AHRQ Health Services Research Projects (R01); PA-13-216: Research On Autism Spectrum Disorders (R01); PA-13-302: Research Project Grant (Parent R01); PA-14-114: Behavioral Interventions To Address Multiple Chronic Health Conditions In Primary Care (R01); PA-14-291: AHRQ Health Services Research Projects (R01); PAR-10-039: Dissemination and Implementation Research In Health (R03); PAR-13-054: Dissemination and Implementation Research In Health (R21); PAR-13-055: Dissemination and Implementation Research In Health (R01); PAR-13-146: NCI Exploratory/Developmental Research Grant Program (NCI Omnibus R21); PAR-16-365: Pilot Clinical Trials For The Spectrum Of Alzheimer’s Disease And Age-Related Cognitive Decline (R01); RM-11-001: Integrating Comparative Effectiveness Research Findings Into Care Delivery Through Economic Incentives (R21)

^b^Not listed = No FOA listed in either the QVR or the NIH RePORT online databases

## Abbreviations

ABIM: American Board of Internal Medicine
AHRQ: Agency for Healthcare Research and Quality
cRCT: Cluster randomized controlled trial
HRT: Hormone replacement therapy
ICs: Institutes and Centers
NCI: National Cancer Institute
NIH: National Institutes of Health
PA: Program announcement
PAR: Program announcement with special receipt, referral, and/or review considerations
PI: Principal investigator
pRCT: Pragmatic randomized controlled trial
PRISMA: Preferred Reporting Items for Systematic Reviews and Meta-Analyses
QVR: Query, View and Report
RCT: Randomized controlled trial
RFA: Request for applications
